# Local modulation of chemoattractant concentrations by single cells: dissection using a bulk-surface computational model

**DOI:** 10.1098/rsfs.2016.0036

**Published:** 2016-10-06

**Authors:** J. A. Mackenzie, M. Nolan, R. H. Insall

**Affiliations:** 1 Department of Mathematics and Statistics, University of Strathclyde, Glasgow G1 1XH, UK; 2 Beatson Institute for Cancer Research, Switchback Road, Bearsden G61 1BD, UK

**Keywords:** chemotaxis, cell motility, self-generated gradients, bulk-surface model, surface finite-elements

## Abstract

Chemoattractant gradients are usually considered in terms of sources and sinks that are independent of the chemotactic cell. However, recent interest has focused on ‘self-generated’ gradients, in which cell populations create their own local gradients as they move. Here, we consider the interplay between chemoattractants and single cells. To achieve this, we extend a recently developed computational model to incorporate breakdown of extracellular attractants by membrane-bound enzymes. Model equations are parametrized, using the published estimates from *Dictyostelium* cells chemotaxing towards cyclic AMP. We find that individual cells can substantially modulate their local attractant field under physiologically appropriate conditions of attractant and enzymes. This means the attractant concentration perceived by receptors can be a small fraction of the ambient concentration. This allows efficient chemotaxis in chemoattractant concentrations that would be saturating without local breakdown. Similar interactions in which cells locally mould a stimulus could function in many types of directed cell motility, including haptotaxis, durotaxis and even electrotaxis.

## 1. Introduction

Cell movement is fundamental throughout medicine and biology. In particular, embryonic development is largely mediated by cells moving relative to one another; immune responses are entirely dependent on white blood cells' amoeboid migration, and cancer metastasis is fuelled by inappropriate movement of tumour cells into the blood, lymph and surrounding tissues. Random movement is an extremely inefficient way to move cells any distance, and limits the ability of cells to explore. Hence, the steering of cell migration by gradients of diffusible chemicals (chemotaxis), and its relatives haptotaxis and durotaxis, is central to moving cells' ability to move between defined sites.

In the mainstream view of chemotaxis described in most of the literature, the gradients of attractants are imposed by external influences, and cells respond relatively passively, simply reading the gradients and moving in response to them. Recently, however, a new paradigm has emerged in which cells have the capability to alter local levels of ligand molecules [1,2]. This can lead to populations of cells generating their own gradients in their local environment. This interaction occurs in various biological contexts. For example, in *Dictyostelium* cells, cyclic AMP (cAMP) is a key chemoattractant that mediates multicellular aggregation. However, cAMP is broken down by secreted and membrane-bound phosphodiesterases; without them, it cannot function [3–5]. *Dictyostelium* cells use an alternative chemoattractant, folate, to locate their bacterial food; folate is broken down, using a dedicated deaminase [6,7]. During zebrafish neural development, the cells of the lateral primordium migrate in a chain that is driven by a self-generated gradient. Migration requires the CXCR7 receptor, which recognizes the chemokine SDF-1 [8]. However, the role of this receptor is not to transduce the SDF-1 signal but to sequester it and hence remove it from the back of the primordium. This leads to a gradient in SDF-1 across the primordium that is actually read and responded to by a separate receptor CXCR4.

Many other types of signalling molecule are used in self-generated gradients. Growth factors, for example—one study shows the ability of epithelial cells to migrate persistently through microscopic mazes that are seeded initially with homogeneous concentrations of epidermal growth factor (EGF). Migration is achieved through the local depletion of EGF, the restricted transport of EGF through the constrained maze structure and the subsequent chemotactic response to the locally self-generated EGF microgradients [9]. Similarly, the lipid signal LPA is a key determinant of melanoma metastasis [10]. Melanoma cells rapidly break down LPA, giving gradients that are low inside and high outside tumours, and provide a steering cue that directs cells out of the tumour.

Because self-generated gradients involve many feedback loops, which can lead to unpredictable behaviour, they are best analysed using mathematical and computational models. The invasion of fibroblast cells in wound healing was considered in [11]. A one-dimensional model was constructed to include the effect of breakdown of platelet-derived growth factor (PDGF), which is both a chemoattractant and a mitogen, through endocytosis of its receptor. The model is shown to predict an invasive wave of cells that dynamically maintain a moderate gradient of PDGF at its leading edge. The invasive wave is robust in the sense that it travels over large length scales where the PDGF concentration varies over orders of magnitude, and is not strongly affected by a range of PDGF secretion rates. In [12], the authors consider a simple one-dimensional model incorporating ligand diffusion, receptor expression and receptor and ligand co-internalization in the vicinity of a moving cell collective. The existence of a dynamically maintained travelling wave solution was established for the coupled system. Furthermore, it was shown that movement of the cell collective results in a higher ligand concentration at the front of the collective compared with that at the rear, thus creating a ligand gradient in the migration direction. This self-generated chemotactic gradient therefore allows the cell collective to migrate over large distances. In [7], an agent-based approach was used to simulate the self-generated chemotaxis of a population of cells. Simulations compared well with experimental data from *Dictyostelium* cells migrating in an under agar assay that was homogeneously seeded with the chemoattractant folate. The agent-based model assumed that individual cells move with a biased random walk with directional persistence arising from an estimate of the difference in receptor occupancy of the individual cells based on the local concentration of the ligand field. Each agent breaks down the ligand, and a linear diffusion model with time-dependent sinks is used to evolve the ligand field in the extracellular region.

While the agent-based approach is flexible and relatively easy to implement computationally, it does not account for important effects such as changes to cell morphology and individual cell polarization. In [13,14], we developed a ‘pseudopod-centred’ [15] model based on a three species reaction–diffusion system involving an autocatalytic local activator, a global inhibitor and a local inhibitor. The read-out level of the local activator was used to drive a simple biomechanical model of forces exerted on the cell membrane by cortical tension and actin polymerization. External signals, where present, steer the cells by slightly biasing their endogenous movement. Using advanced numerical techniques to solve the coupled biochemical and biomechanical system equations, the computational model was remarkably successful in capturing multiple aspects of real cell behaviour including persistent cell migration in the absence of directional signals and chemotaxis in shallow and steep gradient fields [14]. The computational framework was extended recently to model the coupling of physical processes in the extracellular region with those taking place on an evolving cell membrane [16]. This required the development of novel numerical techniques to solve the resulting bulk-surface system of partial differential equations (PDEs). In this paper, we couple the pseudopod-centred model to enzymatic local degradation of chemoattractants to study the ability of single cells, not the populations used in previous work, to affect their own steering by breaking down attractants. Previous studies of self-generated gradients typically consider populations of cells. By fitting parameters to our equations using published estimates from *Dictyostelium* responding to cAMP, we ensure that these studies are physiologically relevant.

The layout of this paper is as follows. In the next section, we introduce the model equations for cell polarization, cell movement and interaction with an extracellular ligand field. This section also includes details of the non-dimensionalization of the model equations and the reference quantities used based on parameter estimates in the literature. In §3, we outline the numerical techniques used to approximate the time-dependent coupled bulk-surface systems arising from the model. The predictions using the computational model are presented in §4. Finally, we make some conclusions and suggest some biological implications of our results and suggestions for future research in §5.

## 2. Methods

### 2.1. Pseudopod-centred model for cell polarization and movement

**Figure 1.**
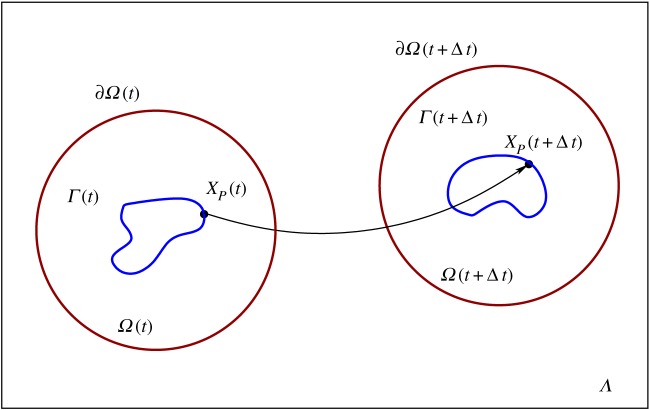
We consider the simulation of a motile cell through a fixed laboratory frame of reference *Λ*. The cell membrane is denoted by *Γ*(*t*) and the extracelluar region close to the cell is denoted by 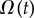 with far-field boundary 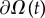. After a time interval of size Δ*t*, the material point located at 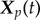 on the cell membrane *Γ*(*t*) evolves to the new location 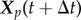.

A schematic of the domains over which the model equations are posed is shown in figure 1. The cell membrane *Γ*(*t*) is assumed to move through a stationary laboratory frame of reference *Λ*. The governing equations for the extracellular region are solved on the evolving region between the cell membrane and a time-dependent circular far-field boundary ∂*Ω*(*t*), which is located a distance *r_f_* from the centroid of the intracellular region enclosed by *Γ*(*t*). We assume that each material point *P* located at *X_p_*(*t*) on *Γ*(*t*), has velocity 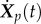. Therefore, there exists a velocity field ***u***, so that points on *Γ*(*t*) evolve such that 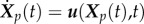.

Let ***n*** = (*n*_1_, *n*_2_) denote the unit outward normal to *Γ*(*t*), and let 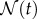 be any open subset of 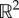 containing *Γ*(*t*). For any function *ζ* which is differentiable in 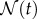, we define the tangential gradient on *Γ*(*t*) by 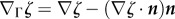, where · denotes the usual scalar product and 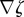 denotes the usual gradient on 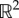. For a vector function 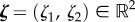, the tangential divergence is defined by

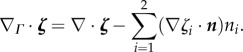


The Laplace–Beltrami operator on *Γ*(*t*) is defined as the tangential divergence of the tangential gradient 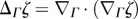.

The following set of equations was derived from a discrete model proposed by Meinhardt [17]. The model describes the interaction between a membrane-bound local autocatalytic activator *a*, a rapidly distributed global inhibitor *b* and a local inhibitor *c*. Assuming that the cell boundary *Γ*(*t*) moves with velocity ***u***, then for 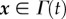 the equations take the form
2.1
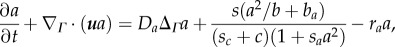

2.2
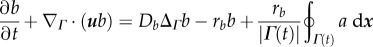

2.3
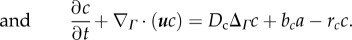


The linear rates of decay of the local activator, global inhibitor and local inhibitor are denoted by *r_a_*, *r_b_* and *r_c_*, respectively. The diffusion coefficients for the three species are denoted by *D_a_*, *D_b_* and *D*_c_. In the activator equation, *s*_*a*_ is a saturation coefficient, *s_c_* is a Michaelis–Menten constant and *b*_*a*_ is a basal production rate of the activator. The rate of growth of the local inhibitor *c* in the presence of the activator *a* is determined by the constant *b_c_*. The signal term *s* incorporates the effect of any external chemotactic field. Owing to the complexity of real cells and the difficulty in obtaining definitive experimental data, at this stage, we do not prescribe specific molecular realizations to the activators and inhibitors in this model. We therefore prefer to view the model as a top-down approach, where each parameter can potentially represent several molecular species. For example, SCAR/WAVE proteins could play the role of the local activator leading to pseudopod actin nucleation [18].

Actin polymerization creates a protrusive pressure that pushes the cell membrane outward in the normal direction. We assume that the rate of polymerization is proportional to the concentration of the local activator. The effect of cortical tension is modelled by a retractive force that is proportional to the local curvature of the membrane. The cell membrane is therefore assumed to evolve according to the geometrical evolution law for the normal velocity
2.4
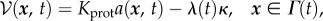
where *K*_prot_ is a positive constant and *κ* denotes curvature. Numerical experimentation with a constant cortical tension coefficient can lead to large unphysical variations in the area enclosed by *Γ*(*t*). We have therefore used a spatially constant but time-dependent cortical tension factor which is the solution of the dynamic equation
2.5
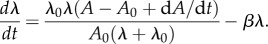
Here, *A*_0_ is the initial area of the cell and *β* and *λ*_0_ are positive parameters. The solution of equation (2.5) is found using an explicit Euler method, and the parameter values for *β* and *λ*_0_ are the same as used in [13].

A more sophisticated model, which includes the effect of the bending rigidity of the membrane, leads to a fourth-order geometric evolution law [19]. Although more faithful to the underlying physics, simulations presented in [19] suggest that there is little qualitative difference in the resulting cell morphologies and behaviour using this model compared with the simpler second-order model (2.4).

### 2.2. Ligand diffusion in the extracellular region

We will assume that the material velocity ***u*** = 0 in the extracellular region *Ω*(*t*) and the concentration of ligand evolves according to the linear diffusion equation
2.6
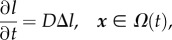
where *l* denotes the ligand concentration and *D* is the extracellular ligand diffusion coefficient. At the far-field boundary, we assume
2.7
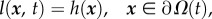
where *h*(***x***) corresponds to a fixed imposed field. At the cell membrane, *Γ*(*t*), we assume that a chemoattractant ligand molecule *L* binds to a receptor *R* at the rate *k*_1_ to form a receptor–ligand complex LR. The complex LR can then disassociate at the rate 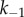 releasing the ligand *L* back off the membrane. We also allow the possibility of the complex LR to diffuse laterally along the membrane. Finally, we also assume that the total concentration of bound and unbound receptors is constant and takes the value *R*_tot_. The concentration of bound receptors, *l_s_*, therefore evolves such that
2.8
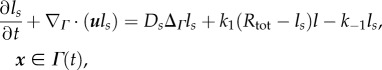
where *D_s_* is the membrane diffusion coefficient.

### 2.3. Enzyme degradation of extracellular ligand field

We now consider extending the model to include membrane-bound enzyme degradation of the extracellular ligand field. We assume that a ligand molecule *L* first binds to a membrane-bound enzyme molecule *E* at the rate *k*_on_ forming an enzyme–ligand complex LE. The complex can disassociate at the rate *k*_off_, or go on to form a product *P* and the original enzyme molecule at the rate *k*_cat_. Assuming a quasi-steady state in the concentration of LE and that the total number of enzyme molecules (bound and unbound) is fixed at *E*_tot_, then it can be shown that
2.9
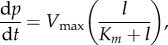
where *p* is the concentration of product, 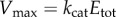 is the maximum rate of degradation at a saturating ligand concentration, and 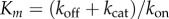 is the Michaelis–Menten constant [20]. A balance of fluxes of ligand molecules at the moving cell membrane is expressed in terms of the normal flux boundary condition
2.10
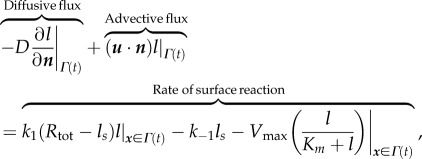
where the cell is advancing into the extracellular region, 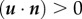, leading to an advective flux onto *Γ*(*t*). Where the cell is retracing away from the extracellular region, 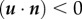, leading to a flux off of *Γ*(*t*). This asymmetry in terms of the advective flux can potentially lead to increased ligand flux at the advancing edge of a cell and less at its receding edge. Cell movement can therefore potentially result in a positive feedback of increased ligand concentration at the cell front thus stabilizing the current direction of motion.

### 2.4. Intrinsic noise

Determining the concentration of bound receptors, *l_s_* allows the estimation of the local fractional receptor occupancy
2.11
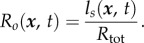
In the absence of any external cues, it has been observed that certain cells move randomly. We therefore include an intrinsic noise component that is independent of the external chemotactic signal. For this purpose, we assume that the intrinsic noise *η^t^* satisfies a stochastic differential equation of mean reverting type [13]. The combined effect of the response to the external signal and random intrinsic noise is modelled by the term

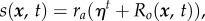
which feeds in multiplicatively to the autocatalytic activator equation (2.1).

### 2.5. Equation non-dimensionalization

For computational purposes, we next non-dimensionalize the coupled bulk-surface system of equations (2.6), (2.8) and (2.10). To do this, we define the non-dimensional variables
2.12
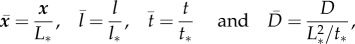
where L_*_ is a characteristic length scale, l_*_ is a characteristic ligand concentration and t_*_ is a characteristic time scale. In terms of the non-dimensional variables, the ligand diffusion equation (2.6) takes the form
2.13
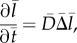
where 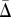 denotes the Laplace operator with respect to the non-dimensional spatial variables. The non-dimensional variables associated with processes at the membrane take the form
2.14
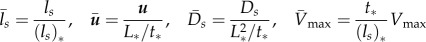
and

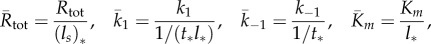
where 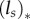 is a characteristic concentration of the ligand–receptor complex. In terms of these variables, equation (2.8) can be written as
2.15
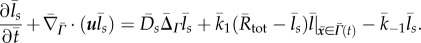


Finally, if 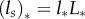, the normal flux condition (2.10) can be expressed as
2.16
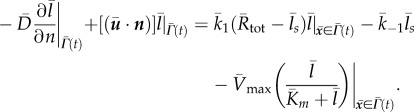


The non-dimensionalized equations therefore take exactly the same form as the original dimensional equations as long as 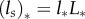.

### 2.6. Choice of reference scales

#### 2.6.1. Time scale

The reference time scale t_*_ is chosen such that the cell speed obtained from the numerical simulations is 10 µm min^−1^ which is approximately the speed of a migrating *Dictyostelium* cell. In the numerical experiments, we have used the reference time scale t_*_ = 1/80 s.

#### 2.6.2. Length scale

The non-dimensional initial radius of the cell in the simulations is 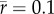. Assuming an initial cell radius *r*_0_ = 5 µm [21], we therefore have a reference spatial scale L_*_ = 50 µm.

#### 2.6.3. Ligand and receptor concentration scales

In the simulations that follow, the reference ligand concentration is l_*_ = 1 nM. For the non-dimensional and the dimensional flux conditions to be equivalent, we therefore set 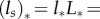
1 nM 50 µm. With the reference scales chosen above, the non-dimensional values for all simulation parameters are therefore specified according to (2.12) and (2.14).

## 3. Numerical solution of model equations

The solution of the model equations poses a considerable computational challenge involving the approximation of nonlinear systems of reaction–diffusion systems on evolving curves coupled to a diffusion equation on an evolving two-dimensional domain. Motivated by the desire to model complex problems in biology and the physical sciences, the numerical solution of bulk-surface PDEs is an area that has received much attention recently. Specific studies include the development and analysis of finite-element discretization methods for steady-state problems on stationary domains [22], and the application of finite-element methods to time-dependent problems on stationary domains [23,24]. The situation is made far more complicated, however, once the bulk and surface domains are time-dependent, and the surface domain is driven by solution components on the surface that are changing dynamically themselves. Here, we outline only the computational techniques used here with detailed descriptions given in [13,16]. The reaction–diffusion system (2.1)–(2.3) is approximated, using an arbitrary Lagrangian evolving finite-element method (ALE–FEM) [25]. The ALE framework is necessary when the time-dependent computational mesh does not necessarily move with the material velocity of individual mesh points. The approximation of the cell membrane is obtained using a novel adaptive moving mesh method that moves mesh points in the normal direction with a velocity determined by the geometrical evolution law (2.4). The method simultaneously moves points in the tangential direction to increase the resolution of solution features or rapid changes to the cell morphology as well as maintaining the overall quality of the mesh. The bulk diffusion equation is approximated using an ALE–FEM method with piecewise linear elements on an evolving triangular mesh. The bulk mesh is generated using an adaptive approach based on the solution of a system of moving mesh partial differential equations (MMPDEs) [26]. Finally, the coupling of the solution components between the bulk region and the cell membrane is achieved, using a predictor–corrector approach based on a second-order Crank–Nicolson time integration scheme.

For the numerical simulations, we have assumed an initial circular cell radius *r*_0_ = 5 µm. The non-dimensional parameter values for the Meinhardt system (2.1)–(2.3) and the mechanical response to the activator level (2.4) are those used in [13,16]. The physical parameters used for the ligand diffusion model, receptor binding–unbinding and enzyme degradation are based on estimates in the literature for *Dictyostelium* cells and are given in table 1.

**Table 1.** Dimensional parameters used in the model of cell migration based on *Dictyostelium discoideum* cells and the ligand cyclic AMP.

quantity	symbol	typical value	
diffusion coefficient of ligand	*D*	4.44 × 10^2^ µm^2^ s^−1^	[27]
diffusion coefficient of receptor–ligand complex	*D_s_*	1 × 10^−1^ µm^2^ s^−1^	[28]
enzyme Michaelis–Menten constant	*K_m_*	0.75 µM	[3]
ligand disassociation rate	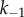	1 s^−1^	[29]
ligand association rate	*k*_1_	1/30 nM^−1^ s^−1^	[29]
number of receptors per cell	*N*_rec_	75 000	[30]
radius of cell	*r*_0_	5 µm	[21]

## 4. Results and discussion

### 4.1. Effect of breakdown

To investigate the effect of enzyme breakdown, simulations were first performed using a stationary circular cell embedded in a linear gradient of chemoattractant. In terms of polar coordinates, the initial ligand concentration is set to
4.1
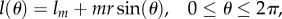
where *l_m_* is the ligand concentration when 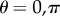 and *m* the gradient. At the far-field boundary, the ligand concentration is kept fixed at its initial value. The initial ligand field at the back of the cell is determined by an imposed equilibrium receptor occupancy *R*_0_, so that

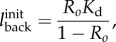
where 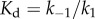 is the receptor disassociation constant. The initial ligand field at the front of the cell is then set to a given percentage increase on that at the back of the cell and this then allows the determination of the linear gradient *m*.

**Figure 2.**
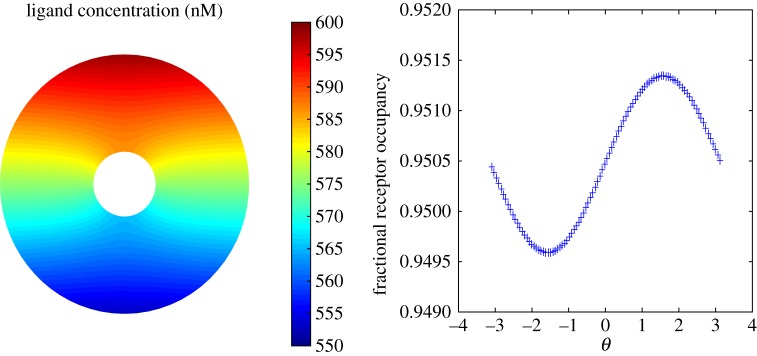
Simulated ligand concentration and receptor occupancy for a stationary circular cell with no breakdown. The far-field concentration corresponds to a saturating field on which is imposed a shallow 2% linear gradient in the ligand concentration from the back to the front of the cell.

**Figure 3.**
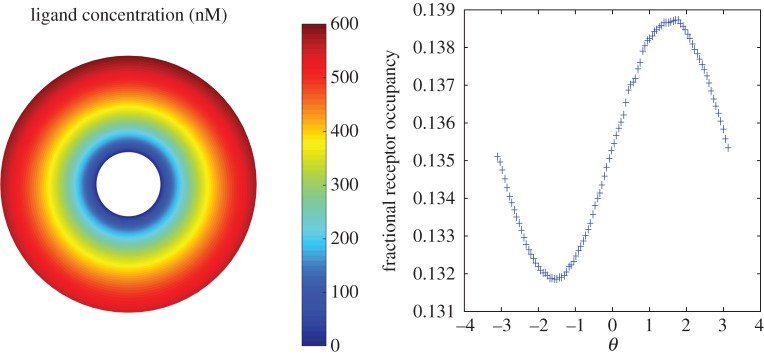
Simulated ligand concentration and receptor occupancy for a stationary circular cell with membrane-bound enzyme breakdown.

Simulations were performed using a saturating ligand concentration which results in 95% of available receptors (*R*_0_ = 0.95) being occupied. A very shallow 2% gradient in the initial ligand concentration from the back to the front of the cell was then imposed. Figure 2 shows the computed steady-state ligand field in the absence of breakdown. We can see that the interaction of the cell membrane receptors alone has a limited effect on the linear field close to the cell. This is due to the inability of the receptors to sequester enough ligand molecules to the cell surface. We see further that the gradient in the receptor occupancy is extremely small, and the absolute value of the receptor occupancy is very close to the initial saturating level. By contrast, figure 3 shows equivalent results when *V*_max_ = 1000 nmoles per 10^7^ cells per minute. We can see that receptor occupancy has been reduced to levels corresponding to a ligand concentration level comparable to the receptor disassociation constant *K*_d_ = 30 nM. Larger values of *V*_max_ lead to significant degradation of the ligand field, so that noise dominates the chemotactic signal. On the other hand, smaller values of *V*_max_ lead to insufficient degradation and receptor saturation and loss of chemotactic efficiency. The value of *V*_max_ used here is somewhat larger than that reported in the literature. For example, Malchow *et al.* [31] find that *V*_max_ = 1.8 nmoles per 10^7^ cells per minute for aggregative stage *Dictyostelium* cells. It is important to point out, however, that the membrane ligand concentration also depends on the extracellular diffusion coefficient. In the simulations presented here, we have used a value in the literature for cAMP diffusing in agar. However, cAMP is multiply charged, so will interact with other charged molecules in its neighbourhood, so its effective diffusion coefficient will be lower. Degradation of the ligand field towards *K*_d_-like levels would then require a far smaller breakdown rate.

### 4.2. Ligand breakdown modulates external chemotactic signals in a saturating environment

**Figure 4.**
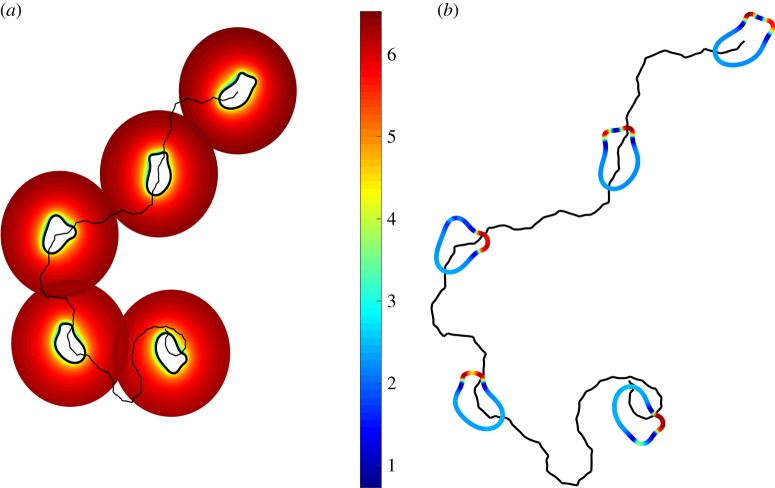
(*a*) Cell migration in an initially saturating linear gradient ligand field. Five snapshots of the position of the cell membrane and ligand field in the extracellular region. Membrane-bound enzyme degradation results in a depletion zone close to the cell. The continuous black line shows the trajectory of the cell centroid and the ligand concentration has been plotted on a log scale. (*b*) Colour plot of the local activator level on the cell membrane.

We next allow the cell to move in the same saturating linear chemotactic field considered above. In the simulations that follow, we set *V*_max_ = 1000 nmol min^−1^ per 10^7^ cells. Figure 4*a* shows five time-lapsed frames of the computed ligand field and position of the cell. We can see that the effect of ligand breakdown and cell movement leads to a narrow depletion zone around the moving cell that displays an elongated morphology with generally two pseudopods driving the migration of the cell at its front. While the cell initially moves in the wrong direction, after a short period it is able to discern the shallow gradient and then directs its movement upgradient. The polarized nature of the local activator concentration driving the cell motion can be seen in figure 4*b*. As seen in multiple real cell types, directed cell migration is a result of a biased selection of pseudopods generated at the front of the cell mainly by a pseudopod splitting mechanism [32].

**Figure 5.**
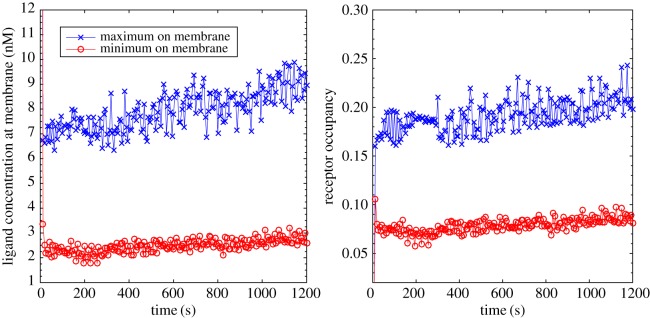
Computed ligand concentration and receptor occupancy on the membrane of an evolving cell in a shallow linear chemotactic field.

The computed ligand concentration and receptor occupancy on the evolving cell membrane are shown in figure 5. We can see that the ligand concentration has been degraded significantly to a level well below the value of *K*_d_. The maximum value occurs at the cell pseudopods, whereas the minimum occurs in proximal lateral regions. There is therefore a significant lateral gradient in the ligand field resulting in a considerable relative difference. We see that the resulting receptor occupancy ranges from around 8% to 20%, and at this level, it is possible for the cell to modulate the generation of pseudopods leading to directed migration.

**Figure 6.**
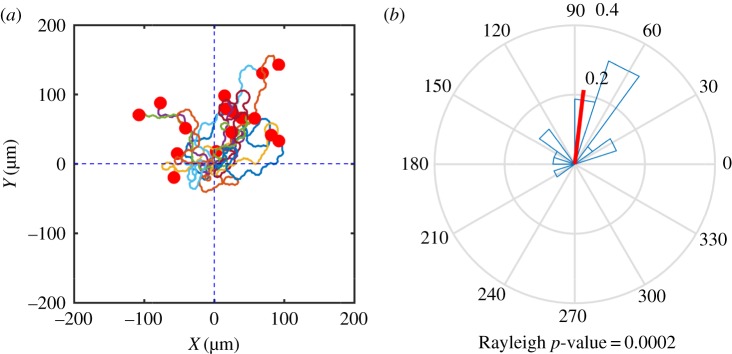
(*a*) Cell trajectories for simulated cells migrating in a shallow linear ligand field. A total of 16 cells are simulated over a time period of 20 min. (*b*) Rose plot of distribution of direction data; the resultant vector is shown in red.

**Figure 7.**
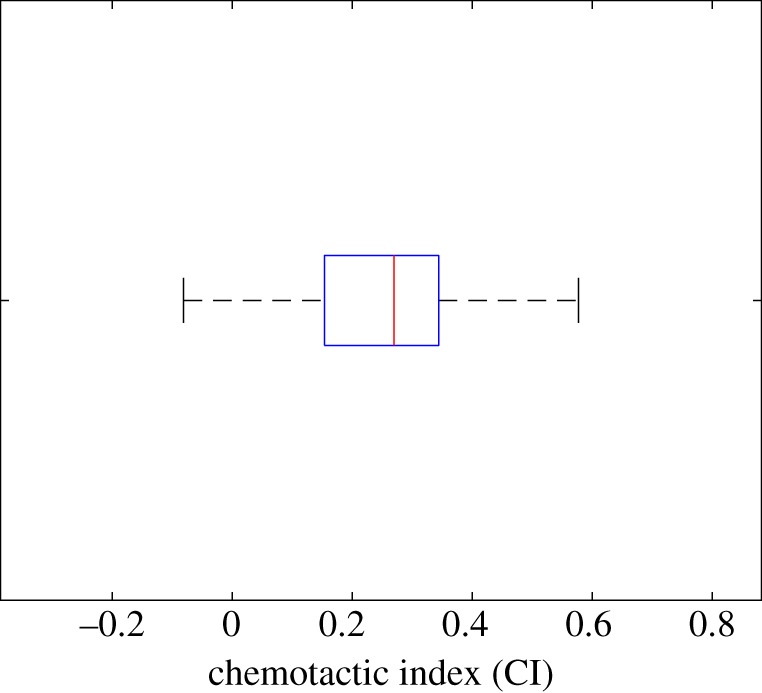
Boxplot of the chemotactic index of 16 simulated cells migrating in an initial saturating linear field of chemoattractant. The mean value 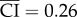 and the standard error of the mean 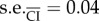.

Figure 6*a* shows the trajectories of the centroids of 16 simulated cells over a time period of 20 min. All of the cells display a biased random walk behaviour, with all but one of them ending up with a net movement in the direction of the chemoattractant gradient. To quantify the directional data, a rose plot of the angle between the straight line joining the initial cell position and the cell centroid at *t* = 20 min is shown in figure 6*b*. The resultant vector of all the cell displacements is shown in red indicating strong evidence of chemotaxis. A Rayleigh test [33] was carried out to investigate the null hypothesis that the population is distributed uniformly around the circle. The test was implemented using the Matlab toolbox CircStat [34]. A calculated value of *p* = 2 × 10^−4^ strongly supports evidence of directional migration. We have also calculated the chemotactic index (CI) of each simulated cell, which is defined here as the ratio of the displacement in the gradient direction to the total length of the cell trajectory. Figure 7 shows a box plot of the distribution of CI. The mean value 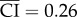 compares well with the experimentally observed value of 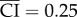 obtained using a similar linear chemotactic field with an initial mean concentration of 500 nM and gradient 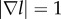 nM µm^−1^ [35].

### 4.3. Breakdown and cell migration in an initially homogeneous ligand field

**Figure 8.**
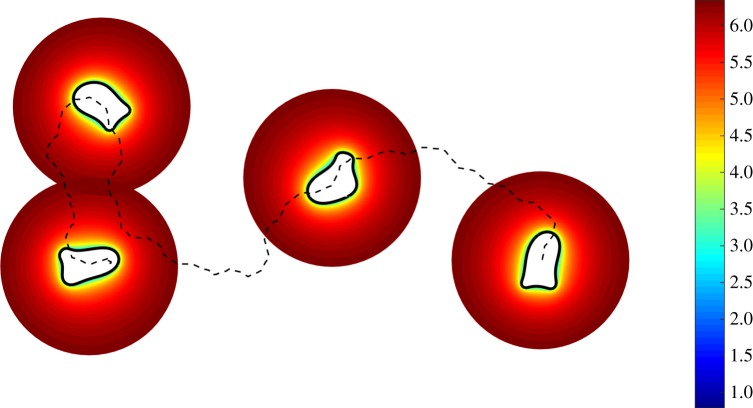
Cell migration in an initially saturating homogeneous ligand field. Four time frames show the position of the cell membrane and ligand field in the extracellular region. Membrane-bound enzyme degradation results in a depletion zone close to the cell. The dotted line shows the trajectory of the cell centroid and the ligand concentration has been plotted on a log scale.

Simulations were performed to test the ability of the modelled cells to migrate in an initially homogeneous ligand field. A saturating concentration *l* = 570 nM was used corresponding to 95% receptor occupancy. Figure 8 shows four snapshots of the computed ligand field in the extracellular region close to a typical migrating cell. We can see that enzyme degradation of the ligand field close to the cell has resulted in a narrow depletion zone where the concentration drops dramatically from the saturating far-field value to a value resulting in a mean receptor occupancy of around 15%. The cell displays undirected persistent cell migration even though the homogeneous far-field concentration would normally lead to receptor saturation.

**Figure 9.**
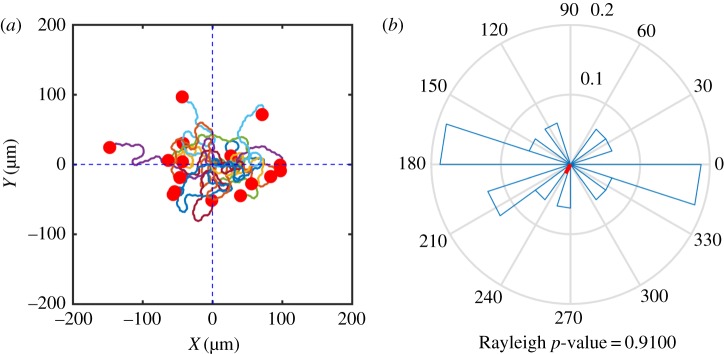
(*a*) Cell trajectories for simulated cells migrating in an initially homogeneous ligand field. A total of 16 cells are simulated over a time period of 20 min. (*b*) Rose plot of distribution of direction data; the resultant vector is shown in red.

Figure 9*a* shows the trajectories of the centroids of 16 simulated cells over a time period of 20 min. All of the cells display a persistent random walk behaviour with no apparent overall directional bias. The rose plot and the resultant vector shown in figure 9*b* indicate that there does not appear to be a preferred mean migration direction. The Rayleigh value *p* = 0.91 suggests that there is no evidence to reject the null hypothesis that the angular information is uniformly distributed.

## 5. Conclusion

We have presented simulations suggesting that single cells can radically change their local chemoattractant levels, in initially saturating environments. Given a correctly tuned degree of breakdown, the ligand field at the membrane can be modulated allowing cell receptors to accurately read off shallow gradients leading to efficient chemotaxis. This has strong implications for the generality of self-generated chemotaxis, as well as the dynamic range of chemotactic responses.

In this work, we have assumed ligand breakdown takes place via the activity of membrane-bound enzymes. Ligand degradation can also be achieved using secreted enzymes. Future work will look at the modelling of these additional mechanisms to determine if there are significant differences in migratory behaviour. In our current model, we have also ignored the effect of receptor internalization and receptor expression. These more realistic assumptions can be included as considered in [36] and it remains to be seen under which circumstances these processes have an effect on self-generated chemotaxis. We also plan to investigate the use of near-field boundary conditions based on Green's functions [37,38] rather than the use of Dirichlet conditions corresponding to an undisturbed field. These should ensure that the computational mesh is not needed to extend far from the evolving cell when diffusion is fast.

The computational model used here has been applied to single cell migration. There is great interest of course on how populations of cells interact, especially when they individually and collectively generate their own chemotactic gradients. We plan to extend the computational framework presented here to investigate the interaction of multiple cells. This will require a procedure for dealing with overlapping computational domains of each individual cell. One possibility is the use of overlapping domain decomposition techniques where each cell can be simulated in parallel thus reducing the overall computational cost. We do believe, however, that the detailed information gained through simulations of single cells or the interaction of a few cells could be used to better inform agent-based approaches and the use of macroscopic models using partial differential equations to evolve cell density fields [39,40]. Currently, such models usually presume that individual cells perceive the concentration of chemoattractant in the bulk medium, in a large-scale gradient. The work we have described shows both presumptions are inaccurate. Taking local breakdown into account, cells may perceive only a small fraction of the bulk attractant concentration, which depending on the level of receptor saturation may make the attractant cause a greater or smaller change in the signal perceived by the cell. Similarly, breakdown may reshape the local steepness of gradients as well as their amplitude. The effect of local attractant breakdown should therefore be considered even in larger-scale models.
